# Supplementary material for: Sleep Quality, Nutrient Intake, and Social Development Index Predict Metabolic Syndrome in the Tlalpan 2020 Cohort: A Machine Learning and Synthetic Data Study
Source: Nutrients. 2024 Feb 23;16(5):612. doi: 10.3390/nu16050612 (PMC10934807; doi:10.3390/nu16050612)
Supplement: Supplementary file 1 [file nutrients-16-00612-s001.zip › nutrients-2841650-supplementary.pdf]

nutrients

MDPI

Article

Sleep Quality, Nutrient Intake, and Social Development Index Predict Metabolic Syndrome in the Tlalpan 2020 Cohort: A Machine Learning and Synthetic Data Study

Guadalupe Gutiérrez-Esparza [1,2,*,†], Mireya Martinez-Garcia [3,†], Tania Ramírez-delReal [4], Lucero Elizabeth Groves-Miralrio [3], Manlio F. Marquez [5], Tomás Pulido [6], Luis M. Amezcua-Guerra [3] and Enrique Hernández-Lemus [7,8,*]

[1] Researcher for Mexico CONAHCYT, National Council of Humanities, Sciences and Technologies, Mexico City 08400, Mexico
[2] Clinical Research, National Institute of Cardiology 'Ignacio Chávez', Mexico City 14080, Mexico
[3] Department of Immunology, National Institute of Cardiology 'Ignacio Chávez', Mexico City 14080, Mexico
[4] Center for Research in Geospatial Information Sciences, Aguascalientes 20313, Mexico
[5] Department of Electrocardiology, National Institute of Cardiology 'Ignacio Chavez', Mexico City 14080, Mexico
[6] Cardiopulmonary Department, National Institute of Cardiology 'Ignacio Chávez', Mexico City 14080, Mexico
[7] Computational Genomics Division, National Institute of Genomic Medicine, Mexico City 14610, Mexico
[8] Center for Complexity Sciences, Universidad Nacional Autónoma de Mexico, Mexico City 04510, Mexico
* Correspondence: ggutierreze@conacyt.mx (G.G.-E.); ehernandez@inmegen.gob.mx (E.H.-L.)
† Co-first authors.

Abstract: This study investigated the relationship between Metabolic Syndrome (MetS), sleep disorders, the consumption of some nutrients, and social development factors, focusing on gender differences in an unbalanced dataset from a Mexico City cohort. We used data balancing techniques like SMOTE and ADASYN after employing machine learning models like random forest and RPART to predict MetS. Random forest excelled, achieving significant, balanced accuracy, indicating its robustness in predicting MetS and achieving a balanced accuracy of approximately 87%. Key predictors for men included body mass index and family history of gout, while waist circumference and glucose levels were most significant for women. In relation to diet, sleep quality, and social development, metabolic syndrome in men was associated with high lactose and carbohydrate intake, educational lag, living with a partner without marrying, and lack of durable goods, whereas in women, best predictors in these dimensions include protein, fructose, and cholesterol intake, copper metabolites, snoring, sobbing, drowsiness, sanitary adequacy, and anxiety. These findings underscore the need for personalized approaches in managing MetS and point to a promising direction for future research into the interplay between social factors, sleep disorders, and metabolic health, which mainly depend on nutrient consumption by region.

Keywords: poor quality sleep; social development index; nutrients; machine learning; features selection; balancing methods; Mexico City; Tlalpan 2020 cohort

check for updates

Citation: Gutiérrez-Esparza, G.; Martinez-Garcia, M.; Ramírez-delReal, T.; Groves-Miralrio, L.E.; Marquez, M.F.; Pulido, T.; Amezcua-Guerra, L.M.; Hernández-Lemus, E. Sleep Quality, Nutrient Intake, and Social Development Index Predict Metabolic Syndrome in the Tlalpan 2020 Cohort: A Machine Learning and Synthetic Data Study. *Nutrients* **2024**, *16*, 612. https://doi.org/10.3390/nu16050612

Academic Editors: Takatoshi Kasai, Ryo Naito and Masahiko Kato

Received: 10 January 2024
Revised: 16 February 2024
Accepted: 19 February 2024
Published: 23 February 2024

Copyright: © 2024 by the authors. Licensee MDPI, Basel, Switzerland. This article is an open access article distributed under the terms and conditions of the Creative Commons Attribution (CC BY) license (https://creativecommons.org/licenses/by/4.0/).

Table S1. Results of logistic regression in women sorted in descending order by coefficient

Variable	Coeficient	P_value
GLU	4.61438598	6.24E-59
TRIG	3.63418178	1.18E-37
WC	1.75532078	2.86E-09
BMI	1.60919304	1.05E-06
SBP	1.40299133	1.15E-12
PROTEI	0.90748897	0.08529715
FRUCT	0.73077934	0.23874313
CHOL_SN	0.72037259	0.06868106
URIC	0.65547784	0.01333401
CU	0.64813271	0.17111299
ST	0.58453044	0.06673992
TAKENAP	0.52306921	0.08428453
TFATAV	0.45378438	0.50511738
SLP6	0.45257216	0.31634152
AFAT	0.40353617	0.39285846
SNORE	0.40096519	0.03765119
SLP9	0.39875326	0.51090309
SMOKING	0.39656832	0.57622716
HEIGHT	0.36843397	0.3602053
VITD	0.34565731	0.19293713
EXSMOKER	0.32652526	0.11109861
INSFB	0.31650888	0.45212319
LACT	0.31406159	0.42909475
AGE	0.26491293	0.17849854
PH	0.26443511	0.68096902
VFAT	0.26247553	0.58626792
MALT	0.20732745	0.50183824
IAT	0.20506743	0.479725
RETINOL	0.18177015	0.68232155
SUCR	0.18008465	0.51158553
STYAWKE	0.1617628	0.54163095
CHOL_ANT	0.15962587	0.71856375
IRON	0.15816576	0.76953296
K	0.15479984	0.81570532
B1	0.11950575	0.83439004
BREATH	0.11727516	0.7376083
VITE	0.11675957	0.70161142
B6	0.08788424	0.86343227
CAFF	0.07777266	0.72971541
TROBLS	0.07165834	0.84037238
SODIUM	0.06499431	0.86254147
POLY	0.06264591	0.85856365
SLPQRAW	0.0603044	0.8127478
SLPSOB1	-0.0185385	0.96462192
CURRENT	-0.01938308	0.97413252
TRAIT_ANX	-0.02819937	0.90004735
MAGN	-0.03600824	0.95688876
SLPOP1	-0.0400532	0.84178799
NA.	-0.04293846	0.8740068
VITC	-0.05132443	0.88725477
LDLCO	-0.05927508	0.8952959

Table S1 continued from previous page

CALOR	-0.13207227	0.87598952
B12	-0.1445629	0.77639742
SOLFB	-0.14880699	0.69465531
CALC	-0.16159519	0.7565454
CRUDE	-0.19893755	0.67623461
STAT_ANX	-0.20092121	0.36918723
SLPNOTQ	-0.21249549	0.41886259
VITK	-0.23625609	0.5414399
SMO_PASS	-0.23627251	0.4685682
ALCOHOL	-0.24089496	0.1195537
ALCO	-0.26775646	0.53730363
SLPA2	-0.2687622	0.20739717
SLPS3	-0.28663189	0.50885402
AWAKEN	-0.34580742	0.24386794
HEMCL	-0.34734947	0.26516726
DROWSY	-0.3479991	0.22112597
ZN	-0.39223322	0.40230761
TOTMET	-0.40208434	0.08124107
MN	-0.47366817	0.10230907
SE	-0.48288291	0.16724211
SLPD4	-0.57325002	0.27193455
GLU_1	-0.60657151	0.29639836
CREA	-0.60735014	0.11428968
SATFAT	-0.61803943	0.34013618
B2	-0.67127121	0.23709781
APROT	-0.72667515	0.16289713
MONFAT	-0.73209194	0.17033049
WEIGHT	-1.0528151	0.00522037
CARBO	-1.26566017	0.0287169
(Intercept)	-3.4369688	5.37E-29
HDLCO	-4.67800035	4.34E-30

Table S2. Results of logistic regression in men sorted in descending order by coefficient

Variable	Coeficient	P_value
GLU	3.94711748	2.45E-39
TRIG	2.98165065	3.25E-24
WC	2.53131848	1.02E-09
IAT	2.06238741	5.13E-11
SBP	1.53063308	1.31E-11
B12	1.41903991	0.00880359
BMI	1.40229014	0.00087404
LACT	1.29691863	0.00581383
CARBO	1.18935354	0.0886463
GLU_1	1.1674073	0.10024746
CU	0.86241521	0.19720812
CRUDE	0.73023815	0.26052964
SE	0.71701752	0.07045393
SOLFB	0.68892284	0.11226852
SLPS3	0.63231119	0.23874266
B6	0.56405727	0.35640401
CAFF	0.55851073	0.03728065

Table S2 continued from previous page

HEIGHT	0.55646599	0.101185
URIC	0.50027773	0.05583058
SLP6	0.49018494	0.42527391
B2	0.471985	0.43641829
TFATAV	0.39187336	0.59775244
SLPSOB1	0.38795715	0.49319732
TROBLS	0.35719897	0.42334118
STAT_ANX	0.31595203	0.29914662
VFAT	0.26279143	0.64973673
NA.	0.22975763	0.42323475
PH	0.2199618	0.74901992
SLPNOTQ	0.20605662	0.54321397
AWAKEN	0.16578114	0.65939078
SLPQRAW	0.15883477	0.66279526
SMOKING	0.13175758	0.86426917
SLPOP1	0.10871661	0.6832381
AFAT	0.07662414	0.87999274
SNORE	0.05505988	0.79733064
SODIUM	0.04176903	0.92002409
EXSMOKER	0.03864623	0.86230051
SMO_PASS	-0.02018857	0.95202882
TAKENAP	-0.02833024	0.94205694
APROT	-0.03904016	0.94254801
B1	-0.03954193	0.95075033
VITC	-0.04315616	0.9232486
VITE	-0.0460136	0.91292523
POLY	-0.04636712	0.91422152
MALT	-0.05420069	0.88162302
VITK	-0.05740398	0.91036994
TOTMET	-0.0581952	0.82892443
MN	-0.07280086	0.8478548
IRON	-0.11372917	0.86265895
TRAIT_ANX	-0.12951548	0.69918749
AGE	-0.13556037	0.58166301
CURRENT	-0.14013162	0.82457946
HEMCL	-0.16034084	0.69891929
K	-0.19400137	0.80012794
ALCOHOL	-0.20071661	0.37849732
CALOR	-0.22980446	0.78894578
CHOL_SN	-0.23654431	0.5734099
SLPD4	-0.25183695	0.71281377
SLPA2	-0.28227907	0.26369716
INSFB	-0.28725811	0.61014935
SLP9	-0.29043375	0.72939731
HDLCO	-0.31881753	0.65211129
ST	-0.34077543	0.36582727
CREA	-0.3831114	0.13099692
BREATH	-0.41692164	0.36786663
MONFAT	-0.44628148	0.45738033
DROWSY	-0.45651035	0.21491969
ALCO	-0.45695453	0.10648652
SUCR	-0.47759461	0.16370051
MAGN	-0.52591754	0.51402708

Table S2 continued from previous page

PROTEI	-0.57765716	0.37021573
CALC	-0.58463238	0.321778
SATFAT	-0.62107309	0.37914123
WEIGHT	-0.63573083	0.24061549
VITD	-0.73962226	0.03904902
STYAWKE	-0.80478525	0.01359924
CHOL_ANT	-0.85126984	0.03210061
LDLCO	-0.91851194	0.03526427
ZN	-0.9776362	0.10372908
RETINOL	-1.16831246	0.03750744
FRUCT	-1.60281909	0.02964749
(Intercept)	-6.91054671	1.44E-33
